# Interrelationship Between Baseline HbA1c, SGLT‐2 Inhibitor Use and Risk of Diabetic Ketoacidosis in Adults With Type 2 Diabetes: A Systematic Review and Meta‐Analysis

**DOI:** 10.1111/dom.70728

**Published:** 2026-04-07

**Authors:** Samuel Seidu, Setor K. Kunutsor, Erwin Taguiam, Kamlesh Khunti

**Affiliations:** ^1^ Leicester Real World Evidence Unit, Diabetes Research Centre University of Leicester, Leicester General Hospital Leicester UK; ^2^ Hockley Farm Medical Practice Leicester UK; ^3^ Section of Cardiology, Department of Internal Medicine, Max Rady College of Medicine, Rady Faculty of Health Sciences University of Manitoba Winnipeg Manitoba Canada

**Keywords:** diabetic ketoacidosis, observational study, randomized controlled trial, SGLT‐2i, type 2 diabetes

## Abstract

**Aims:**

There is ongoing uncertainty about whether initiating sodium–glucose co‐transporter 2 inhibitor (SGLT‐2i) therapy in individuals with type 2 diabetes (T2D) who have elevated baseline HbA1c levels may lead to an additive or potentially synergistic increase in the risk of diabetic ketoacidosis (DKA). This systematic review and meta‐analysis aimed to investigate the interrelationship between baseline HbA1c, SGLT‐2i use and the risk of DKA in adults with T2D.

**Materials and Methods:**

Observational cohort studies and randomized controlled trials (RCTs) comparing SGLT‐2is with placebo or active comparators in adults with T2D that reported baseline HbA1c and DKA events were identified through searches in MEDLINE, Embase, CENTRAL, ClinicalTrials.gov and bibliographies up to January 2026. Key characteristics of the study design, patients, interventions and outcomes were extracted. Risk of bias was evaluated. Risk ratios (RRs) were pooled and stratified by HbA1c (high vs. < low). Effect modification was assessed using random‐effects meta‐regression.

**Results:**

Twenty‐two studies (15 cohorts, 7 RCTs) were included. SGLT‐2is were associated with increased DKA risk overall. In observational studies, risk was higher in those with elevated HbA1c (RR 1.63, 95% CI 1.46–1.81) but not in those with lower HbA1c (RR 1.10, 0.80–1.51), with significant effect modification (*p* = 0.018). In RCTs, pooled RRs were 2.37 (1.44–3.90) and 2.01 (0.84–4.79) in high and low HbA1c groups, respectively, without significant interaction (*p* = 0.73). Elevated HbA1c was independently associated with DKA among SGLT‐2i users (RR 1.50, 1.17–1.92). Evidence certainty ranged from high to low.

**Conclusions:**

SGLT‐2i use is associated with an increased risk of DKA in adults with T2D, with a higher risk observed in those with elevated baseline HbA1c, particularly in real‐world settings. However, the narrow range of HbA1c values across studies limits definitive conclusions, and further research is warranted.

Trial Registration: https://www.crd.york.ac.uk/PROSPERO/view/CRD420251149386: CRD420251149386

## 1 Introduction

Diabetes (with type 2 diabetes mellitus (T2D) making up the majority of diabetes cases) represents one of the most pressing global health challenges, currently affecting over 500 million individuals worldwide [1]. Its increasing prevalence is driven by population aging, sedentary lifestyles and the global obesity epidemic. Beyond hyperglycemia, T2D is associated with a substantial risk of microvascular and macrovascular complications, including cardiovascular disease, stroke, kidney failure and peripheral arterial disease, resulting in high rates of morbidity, mortality and health system burden [2].

The comprehensive management of T2D requires not only optimal glycemic control but also aggressive modification of other cardiometabolic risk factors, including blood pressure and lipid levels [2]. In recent years, novel classes of antidiabetic agents, namely sodium‐glucose cotransporter‐2 inhibitors (SGLT‐2is) and glucagon‐like peptide‐1 receptor agonists (GLP‐1RAs), have transformed diabetes care. Landmark cardiovascular outcome trials (CVOTs) have shown that both classes of agents demonstrate potent glucose‐lowering effects and significant reductions in cardiovascular and kidney events [3, 4, 5]. Notably, the benefits of SGLT‐2is extend beyond glycemic control, with clinical trials showing reduced risks of hospitalization for heart failure and progression of chronic kidney disease [3, 4]. These findings have broadened the indications for SGLT‐2is, making them central to the management of patients with T2D and comorbid cardiovascular or kidney disease. However, alongside their benefits, concerns have emerged regarding the safety profile of SGLT‐2is, particularly in relation to diabetic ketoacidosis (DKA). Though rare, DKA is a serious, potentially life‐threatening metabolic emergency marked by hyperketonemia and metabolic acidosis [6]. Poor glycemic control, typically reflected in elevated baseline glycated haemoglobin (HbA1c) levels, has been independently associated with a higher likelihood of DKA [7]. In the context of SGLT‐2i use, DKA may present with normal or only mildly elevated blood glucose levels, a phenomenon termed euglycemic DKA [8]. This atypical presentation is attributable to SGLT‐2i–induced glycosuria, which lowers circulating glucose concentrations despite ongoing ketogenesis. The pathophysiology involves relative insulin deficiency, increased glucagon secretion, enhanced lipolysis and hepatic ketone production, reduced renal ketone clearance and suppression of overt hyperglycemia [8]. Importantly, euglycemic presentation complicates clinical recognition and may delay treatment, but it does not negate the contribution of poor baseline glycemic control as a risk factor. A meta‐analysis of randomized controlled trials (RCTs) revealed that patients using SGLT‐2is had a 2.5‐fold increased risk of DKA compared to those on placebo or other antidiabetic agents [9]. Similarly, evidence from 14 primarily noncommercial, active comparator/new user (ACNU) cohort studies estimated a 33% elevated risk associated with SGLT‐2i use [10]. Clinicians are therefore understandably cautious when initiating SGLT‐2i therapy in patients with markedly elevated HbA1c levels, given concerns about a potentially additive or even synergistic increase in DKA risk. Despite this clinical hesitation, the nature of the interplay between baseline HbA1c and SGLT‐2i use in determining DKA risk remains unclear and underexplored in the literature.

This systematic review and meta‐analysis aims to address this important clinical gap by comprehensively evaluating the interrelationship between baseline HbA1c, SGLT‐2i use and the risk of DKA in adults with T2D. Specifically, it explores whether HbA1c modifies the strength or direction of the association between SGLT‐2i use and DKA, and whether higher baseline HbA1c is associated with increased DKA risk among SGLT‐2i users.

## 2 Methods

### 2.1 Data Sources and Search Strategy

This systematic review and meta‐analysis was conducted based on a predefined protocol registered with PROSPERO (CRD420251149386). The methodology adhered to the PRISMA (Preferred Reporting Items for Systematic Reviews and Meta‐Analyses) and MOOSE guidelines, as outlined in Supporting Information S1 and S2. Comprehensive literature searches were performed in MEDLINE (Ovid), Embase (Ovid) and the Cochrane Central Register of Controlled Trials (CENTRAL, Wiley) from database inception through January 2026. Search strategies combined controlled vocabulary and free‐text terms related to ‘type 2 diabetes’, ‘HbA1c’, ‘SGLT‐2 inhibitors’ and ‘diabetic ketoacidosis’. There were no language restrictions. All retrieved records were imported into Covidence (Melbourne, Australia) for automatic deduplication and screening. Detailed search strategies are provided in Supporting Information S3, and reporting of the search process followed the PRISMA‐S extension for transparent documentation of systematic review searches (Supporting Information S4). To ensure the thoroughness of our search strategy and to avoid missing any relevant studies, we implemented several supplementary strategies. These included (i) searching ClinicalTrials.gov to identify secondary publications and outcome reports related to relevant trials, (ii) manually screening the reference lists of key studies and pertinent review articles, (iii) conducting forward citation tracking of eligible studies and related reviews using the ISI Web of Science database and (iv) performing detailed forward and backward snowballing to capture any additional potentially eligible publications. Screening and data management were facilitated using the Covidence platform. Title and abstract screening were independently performed by two reviewers (E.T. and S.K.K.). Discrepancies were resolved through discussion with a third reviewer (S.S.). Full‐text evaluation was undertaken independently by two reviewers (E.T. and S.K.K.), with any disagreements also resolved through consultation with the third reviewer.

### 2.2 Study Selection and Eligibility Criteria

Eligible studies included RCTs and comparative observational cohort studies (*N* ≥ 500 patients receiving SGLT‐2is) [9] that compared SGLT‐2i use with placebo or other active comparators in adults with T2D and provided both baseline HbA1c values and data on DKA incidence. For RCTs, only those with a minimum duration of 12 weeks were considered eligible to ensure adequate exposure and follow‐up time. We deliberately restricted inclusion to observational studies employing rigorous pharmacoepidemiologic designs—specifically ACNU or prevalent new‐user frameworks—which are widely recommended to minimize confounding by indication, immortal time bias and biases related to prior treatment exposure. These designs more closely emulate the structure of randomized trials by comparing initiators of alternative therapies within similar clinical contexts. To further enhance internal validity and comparability between treatment groups, we required that observational studies implement 1:1 propensity score matching or equivalent high‐quality adjustment strategies to control for measured confounding. This approach was intended to maximize methodological robustness and reduce the risk of biased effect estimates. Additionally, we included observational cohort studies with a minimum sample size of 500 participants that evaluated the association between baseline HbA1c levels and the risk of DKA in adults with T2D treated with SGLT‐2is. We excluded studies that focused on individuals with type 1 diabetes. Studies that reported zero DKA events in either the intervention or comparator arm were excluded. The primary outcome of interest was the incidence or relative risk of DKA.

### 2.3 Data Extraction

For this review, we used a structured and piloted data extraction form. Two reviewers (E.T. and S.K.K.) independently extracted data from each included study. Any discrepancies or disagreements were resolved through discussion and consensus. If necessary, a third reviewer was available to adjudicate unresolved issues. For each included study, we collected publication‐level details such as the year of publication and the geographical region or country in which the study was conducted. We also extracted key characteristics of the study design, including the type of study (e.g., RCT or observational cohort), total sample size, duration of follow‐up and methodological features relevant to internal validity. For RCTs, we documented information on random sequence generation, allocation concealment, blinding of participants and outcome assessors and the duration of the intervention. Participant‐level characteristics included the mean or median age of study participants, the proportion of male and female participants, the duration of T2D at baseline (where reported) and baseline mean or median HbA1c levels. Detailed information was extracted on the intervention and comparator groups. This included the specific SGLT‐2i used, dosage and duration of treatment. For observational studies, we noted whether an ACNU or prevalent new user design was employed. We also captured information on the characteristics of the comparator group, such as whether it involved placebo, another glucose‐lowering medication, or standard of care. Where available, we extracted data on baseline insulin use. The rationale for including this variable is based on its relevance as a marker of endogenous insulin reserve and reliance on exogenous insulin for glycemic control and suppression of ketogenesis. In individuals with high HbA1c, insulin requirements are often elevated and SGLT‐2is can exacerbate the risk of ketosis by promoting glycosuria‐induced reductions in insulin dosing, enhanced glucagon secretion and a lowered insulin‐to‐glucagon ratio [6]. Stratifying analyses by baseline insulin use may therefore help elucidate whether insulin‐treated individuals are disproportionately affected by the DKA risk associated with SGLT‐2is, particularly in the context of elevated HbA1c levels. We recorded the number of DKA events reported in each group, as well as the effect estimates (hazard ratios, risk ratios, or odds ratios) and their corresponding 95% confidence intervals (CIs).

### 2.4 Risk of Bias and Certainty of Evidence

We assessed the risk of bias for each study. For observational studies, we used the Risk of Bias In Non‐randomized Studies of Interventions (ROBINS‐I V2) tool, which assesses potential sources of bias across domains such as confounding, selection, classification of interventions and outcome measurement. For RCTs, we employed the latest version of the Cochrane risk of bias tool (ROB2). Risk of bias assessments were visualized using the robvis web app [11]. To formally appraise the credibility of subgroup effects, we applied the ICEMAN (Instrument to assess the Credibility of Effect Modification Analyses) framework. This tool evaluates key domains including pre‐specification of subgroup hypotheses, biological plausibility, consistency across studies and statistical evidence of interaction [12]. The ICEMAN assessment was used to guide interpretation of the HbA1c‐based effect modification analyses. Given that ICEMAN was primarily developed for RCTs, findings from observational studies were interpreted using the same conceptual domains, with explicit consideration of ecological bias and residual confounding. Finally, we used the Grading of Recommendations Assessment, Development and Evaluation (GRADE) framework to assess the overall certainty of the evidence for each outcome. This approach incorporates considerations such as study limitations (risk of bias), inconsistency of results across studies, imprecision of effect estimates, indirectness of evidence and the potential for publication bias.

### 2.5 Statistical Analysis

The primary summary measure of effect was the risk ratio (RR) with 95% CIs. For the pooled analysis on the association between HbA1c and DKA risk among SGLT‐2i users with T2D, risk estimates were transformed to a top versus bottom quantile using standard statistical methods described previously [13] to enhance comparison and interpretation of the findings. Risk ratios were combined using random‐effects models to minimize the effect of between‐study heterogeneity. Statistical heterogeneity among studies was assessed using both the Cochrane *χ* ^*2*^ statistic and the *I* ^*2*^ statistic. The *χ* ^2^ test evaluates whether observed differences in results are compatible with chance alone, while the *I* ^2^ statistic quantifies the proportion of total variation across studies due to true heterogeneity rather than sampling error, with values of 25%, 50%, and 75% considered low, moderate and high heterogeneity, respectively. We tested for effect modification by baseline HbA1c levels (high: ≥ 8.30% vs. low: < 8.30% categories) using stratified analysis and random‐effects meta‐regression. Because this review was based on aggregate published data rather than individual participant data, HbA1c categories could not be pre‐specified using clinically defined thresholds. The cut‐off was therefore determined based on the distribution of mean baseline HbA1c values reported across the included studies. This approach allowed for an approximately balanced representation of studies in each category, thereby preserving statistical power and facilitating interpretable stratified and meta‐regression analyses. In our pre‐specified protocol, we intended to: (i) explore for sources of heterogeneity using stratified analysis and random effects meta‐regression and (ii) assess for small study effects (e.g., publication bias) using formal tests such as Begg's funnel plots and Egger's regression symmetry test. However, these steps could not be undertaken because each outcome relied on aggregated analyses from fewer than 10 studies. All statistical tests were two‐sided, and a *p*‐value of ≤ 0.05 was considered indicative of statistical significance. All analyses were conducted using Stata MP version 18 (StataCorp, College Station, TX).

## 3 Results

### 3.1 Study Identification and Selection

**FIGURE 1:**
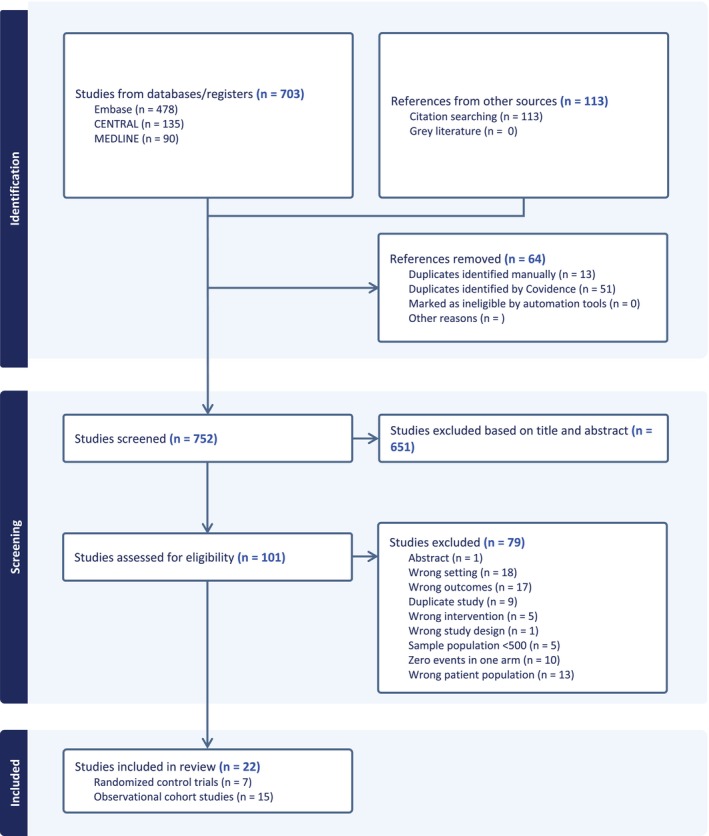
Selection of studies included in the meta‐analysis.

Figure 1 illustrates the study selection process. Searches of MEDLINE, Embase and CENTRAL identified 703 records, with an additional 113 records retrieved through other sources, including citation searching. After removal of duplicates, 752 unique records remained for title and abstract screening. Of these, 651 were excluded at the screening stage as they did not meet the predefined eligibility criteria. The remaining 101 articles underwent full‐text assessment, after which 79 were excluded for specified reasons. Ultimately, 22 studies met all inclusion criteria and were included in the review [14, 15, 16, 17, 18, 19, 20, 21, 22, 23, 24, 25, 26, 27, 28, 29, 30, 31, 32, 33, 34, 35].

### 3.2 Study Characteristics and Risk of Bias

Observational Studies—Table 1 provides details of the key characteristics of 15 observational cohort studies included in the review. Twelve studies compared SGLT‐2i use with non‐SGLT‐2is in adults with T2D and provided extractable data relevant to this meta‐analysis, including baseline HbA1c values and DKA incidence (total participants = 1 269 005; SGLT‐2i group = 495 854; and non‐SGLT‐2i group = 773 151). The mean ranges for baseline age, baseline HbA1c and follow‐up durations were 56.8–72.0 years, 6.90%–8.99% and 0.4–3.1 years, respectively. With the exception of one study, in which 88.7% of participants in the SGLT‐2i group were receiving insulin at baseline, the proportion of insulin‐treated patients across the remaining studies was low and ranged from 7.6% to 32.4%.

**TABLE 1:** Baseline characteristics eligible studies (2015–2025).

Author, year of publication	Study/databases/registries	Baseline year	Males, %	Mean age, years	Mean/median HbA1c %	Location	Follow‐up, years	Intervention (*n*)	Comparator (*n*)	Total sample size
Randomized controlled trials										
Zinman 2015	EMPA‐REG OUTCOME	2010–2013	71.5	67.1	8.07	42 countries	3.1	Empagliflozin (4687)	Placebo (2333)	7020
Neal 2017	CANVAS Program	2009, 2014	64.2	63.3	8.02	30 countries	3.6	Canagliflozin (5795)	Placebo (4347)	10 142
Cannon 2020	VERTIS CV	2013–2015/2016–2017	70.0	64.4	8.20	34 countries	3.5	Ertugliflozin (5499)	Placebo (2747)	8246
Bhatt 2021a	SCORED	2017–2020	55.1	69.0	8.30	44 countries	1.3	Sotagliflozin (5292)	Placebo (5292)	10 584
Wiviott 2019	DECLARE‐TMI 58	2013–2018	62.6	63.9	8.30	33 countries	4.2	Dapagliflozin (8582)	Placebo (8578)	17 160
Perkovic 2019	CREDENCE	2014–2017	66.1	63.0	8.30	34 countries	2.6	Canagliflozin (2202)	Placebo (2199)	4401
Bhatt 2021b	SOLOIST‐WHF	2018–2020	66.3	70.0	7.10	32 countries	0.8	Sotagliflozin (608)	Placebo (614)	1222
Observational Studies										
McGurnaghan 2019	SCIDiabetes	2004–2016	56.5	65.8	7.57	Scotland	0.6	Dapagliflozin (8516)	Never‐users (230360)	238 876
Fralick 2021	ICES drug database	2015–2019	60.0	72.0	8.15	Canada	1.1	SGLT‐2i (29916)	DPP‐4i (29916)	59 832
Patorno 2021	Optum's Clinformatics, IBM MarketScan and Medicare	2013–2017	48.9	60.8	8.95	USA	0.6	Canagliflozin, dapagliflozin, orempagliflozin (186040)	GLP‐1RA (186040)	372 080
Lugner 2021	NDR	2013–2017	62.5	60.6	8.30	Sweden	1.4	Empagliflozin, dapagliflozin, canagliflozin (12097)	GLP‐1RA (9684)	21 781
Fralick 2021b	Optum Clinformatics Data Mart	2013–2017	55.7	57.3	8.70	USA	0.5	Empagliflozin, canagliflozin, dapagliflozin (111442)	—	111 442 (475)^a^
Goh 2022	MOH administrative database	2015–2018	54.7	56.8	7.61	Singapore	0.6	SGLT‐2i (15207)	DPP‐4i (15207)	30 414
Güdemann 2024	CPRD	2013–2020	60.4	61.1	8.99	UK	1.8	Canagliflozin, dapagliflozin, empagliflozin, ertugliflozin (77229)	DPP‐4i (109606)	186 835
Jing 2024	The Hong Kong Diabetes Study	2015–2020	59.5	57.9	NA	Hong Kong	5.5	SGLT‐2i (28814)	NA	28 814 (494)^a^
Htoo 2024	EMPRISE	2014–2019	55.1	62.5	8.95	USA	0.4	Empagliflozin (115116)	DPP‐4i (115116)	230 232
Edmonston 2024	PCORnet	2016–2020	50.4	61.0	8.00	USA	1.1	Empagliflozin (20279)	DPP‐4i (41918)	62 197
Pan 2024	TriNetX US Collaborative Network	2002–2022	59.8	64.0	8.35	USA	2.3	SGLT‐2i (5317)	SGLT‐2i nonuser (5317)	10 634
Yen 2024	NHIRD	2016–2021	50.2	61.1	8.20	Taiwan	3.1	SGLT‐2i (23854)	SGLT‐2i nonuser (23892)	47 746
Chen 2025	CGRD	2016–2022	53.1	67.9	7.50	Taiwan	1.1	Canagliflozin, dapagliflozin and empagliflozin (1012)	DPP‐4i (1012)	2024
Suzuki 2025	DeSC database	2014–2021	60.4	68.0	6.90	Japan	1.7	Empagliflozin, dapagliflozin, canagliflozin, ipragliflozin, tofogliflozin, luseogliflozin, combination (1271)	DPP‐4i (5083)	6354
Tsur 2025	CHS	2015–2022	64.4	53.3	9.00	Israel	4.4	SGLT‐2i (6572)	SGLT‐2i non‐users (6382)	12 954 (239)^a^

Abbreviations: CANVAS Program, CANagliflozin cardioVascular Assessment Study; CGRD, Chang Gung Research Database; CHS, Clalit Health Services; CPRD, Clinical Practice Research Datalink; CREDENCE, Evaluation of the Effects of Canagliflozin on Renal and Cardiovascular Outcomes in Participants With Diabetic Nephropathy; DECLARE‐TMI 58, Multicenter Trial to Evaluate the Effect of Dapagliflozin on the Incidence of Cardiovascular Events; DeSC database, DeNA Co. Ltd. and Sumitomo Corporation Healthcare database; DPP‐4i, dipeptidyl peptidase‐4 inhibitor; EMPA‐REG OUTCOME, (Empagliflozin) Cardiovascular Outcome Event Trial in Type 2 Diabetes Mellitus Patients; EMPRISE, The EMPagliflozin compaRative effectIveness and SafEty cohort study; GLP‐1RAs, glucagon‐like peptide‐1 receptor agonists; HbA1c, glycated haemoglobin; ICES drug database, Institute for Clinical Evaluative Sciences; MOH administrative database, Singapore Ministry of Health administrative database; NDR, The Swedish National Diabetes Register; NHIRD, Taiwan's National Health Insurance Research Database; PCORnet, The National Patient‐Centred Clinical Research Network; SCIDiabetes, Scottish Care Information‐Diabetes; SCORED, Effect of Sotagliflozin on Cardiovascular and Renal Events in Participants With Type 2 Diabetes and Moderate Renal Impairment Who Are at Cardiovascular Risk; SGLT‐2is, sodium–glucose co‐transporter‐2 inhibitors; SOLOIST‐WHF, Effect of Sotagliflozin on Cardiovascular Events in Participants With Type 2 Diabetes Post Worsening Heart Failure; VERTIS CV, Cardiovascular Outcomes Following Ertugliflozin Treatment in Type 2 Diabetes Mellitus Participants With Vascular Disease.

^a^ Represents total participants (No. of DKA events).

Three studies specifically evaluated the association between HbA1c and DKA risk in patients with diabetes receiving SGLT‐2is (153 210 participants, 1208 DKA events). Two of the studies were specifically based on patients with T2D and the third was based on patients with insulin‐deficient diabetes phenotype. Four studies were at serious risk of bias (i.e., were judged to be at serious risk of bias in at least one domain) and the rest were at moderate risk of bias (Supporting Information S5).

Randomized controlled trials—Seven RCTs that compared SGLT‐2i use with placebo in adults with T2D and provided both baseline HbA1c values and data on DKA incidence were included in the review (Table 1). Altogether, they comprised a total of 58 761 participants (297 DKA events) (SGLT‐2i group = 32 656; placebo group = 26 105). The mean ranges for baseline age, baseline HbA1c, treatment duration and follow‐up durations were 63.0–70.0 years, 7.1%–8.3%, 0.7–4.2 years and 0.8–4.2 years, respectively. Across RCTs, the proportion of patients on baseline insulin in the SGLT‐2i group ranged from 35.7% to 65.9%; in patients with higher baseline HbA1c (≥ 8.30%), baseline insulin use was higher (41.6% to 65.9%) compared to those with lower baseline HbA1c (< 8.30%) (35.7% to 49.9%). All included trials were judged to have a low risk of bias across all evaluated domains (Supporting Information S6).

### 3.3 SGLT‐2is and DKA Risk Stratified by HbA1c Levels in Observational Studies

**FIGURE 2:**
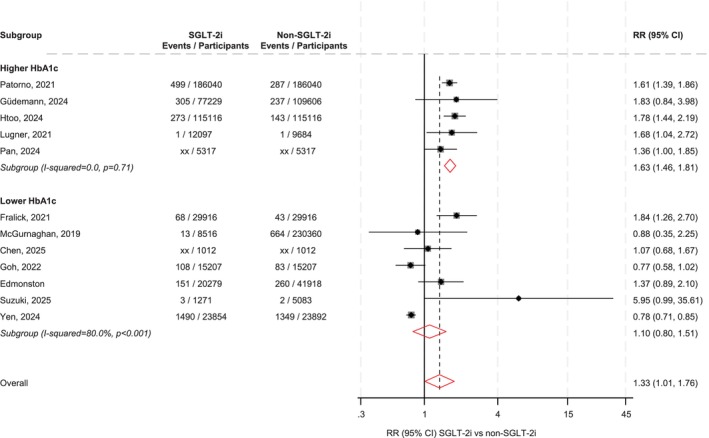
SGLT‐2is and DKA risk overall and stratified by HbA1c levels in observational studies. CI, confidence interval (bars); DKA, diabetic ketoacidosis; HbA1c, glycated haemoglobin; RR, risk ratio; SGLT‐2i, sodium–glucose co‐transporter 2 inhibitor.

In observational studies, SGLT‐2i use was associated with a higher risk of DKA compared with non‐use (pooled RR = 1.33; 95% CI: 1.01–1.76) (Figure 2). Stratified analysis demonstrated a significantly increased risk of DKA among individuals with higher baseline HbA1c (≥ 8.30%) (RR = 1.63; 95% CI: 1.46–1.81), whereas no significant association was observed in those with lower baseline HbA1c (< 8.30%) (RR = 1.10; 95% CI: 0.80–1.51). Meta‐regression confirmed a significant difference in DKA risk between HbA1c categories (*p* = 0.018).

### 3.4 SGLT‐2is and DKA Risk Stratified by HbA1c Levels in RCTs

**FIGURE 3:**
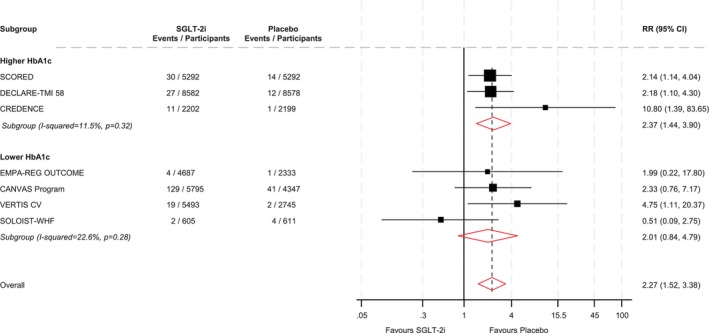
SGLT‐2is and DKA risk overall and stratified by HbA1c levels in randomized controlled trials. CI, confidence interval (bars); DKA, diabetic ketoacidosis; HbA1c, glycated haemoglobin; RR, risk ratio; SGLT‐2i, sodium–glucose co‐transporter 2 inhibitor.

In the RCTs, overall patients allocated to a SGLT‐2i vs. a non‐SGLT‐2i had a higher risk of DKA 2.27 (95% CI: 1.52–3.38) (Figure 3). When stratified by baseline HbA1c, the pooled RR for DKA was 2.37 (95% CI: 1.44–3.90) in the high HbA1c group and 2.01 (95% CI: 0.84–4.79) in the low HbA1c group, with no significant effect modification by HbA1c observed (meta‐regression *p* = 0.73) (Figure 3).

In a subgroup analysis stratified by the proportion of insulin‐treated patients in the SGLT‐2i group, there was no significant evidence of effect modification. The pooled RR for DKA was 2.49 (95% CI: 1.38–4.51) in studies with a high proportion of insulin users (≥ 50.0%), and 2.02 (95% CI: 0.97–4.21) in those with a low proportion of insulin users (< 50.0%) (meta‐regression *p* = 0.63; Supporting Information S7).

In pooled analysis of three observational cohort studies that evaluated the association between HbA1c and DKA risk in patients with T2D receiving SGLT‐2is, the multivariable‐adjusted RR (95% CI) of DKA comparing high vs. low HbA1c categories was 1.50 (95% CI: 1.17–1.92) (Supporting Information S8).

### 3.5 Credibility of Subgroup Results (ICEMAN Assessment)

Using the pre‐specified ICEMAN framework to evaluate the credibility of the observed effect modification by baseline HbA1c, the subgroup hypothesis was judged to be biologically plausible and specified a priori. In observational studies, statistical evidence supported effect modification; however, this was not replicated in the randomized trial data. Given the limited number of contributing studies, reliance on aggregate study‐level data and inconsistency across study designs, the overall credibility of the subgroup effect was considered moderate in observational analyses and low in RCTs (Supporting Information S9).

### 3.6 GRADE Summary of Findings

The following outcomes were selected for GRADE evaluation: (i) SGLT‐2is and DKA risk in higher HbA1c group for observational studies; (ii) SGLT‐2is and DKA risk in lower HbA1c group for observational studies; (iii) SGLT‐2is and DKA risk in higher HbA1c group for RCTs; and (iv) SGLT‐2is and DKA risk in lower HbA1c group for RCTs. GRADE certainty of the evidence ranged from moderate to low for observational studies (Supporting Information S10) and high to moderate for RCTs (Supporting Information S11).

## 4 Discussion

This systematic review and meta‐analysis provide comprehensive evidence on the interrelationship between baseline HbA1c levels, SGLT‐2i use and the risk of DKA in adults with T2D. First, aggregate data from 15 observational studies and 7 CVOTs demonstrated that SGLT‐2is, compared with non‐SGLT‐2i therapies, are associated with a significantly increased risk of DKA overall. Second, stratified analyses of observational studies revealed that this increased risk was driven primarily by individuals with high baseline HbA1c, in whom the association was statistically significant. In contrast, no significant association was observed in individuals with low HbA1c. Importantly, meta‐regression confirmed significant effect modification by HbA1c, suggesting that baseline glycemic control may influence DKA risk in real‐world settings. Third, in the randomized trial data, stratified analyses showed a similar pattern: a significantly increased risk of DKA in participants with high HbA1c, but not in those with low HbA1c. However, meta‐regression did not demonstrate statistically significant effect modification by HbA1c levels in the RCTs. Although the subgroup hypothesis was pre‐specified and biologically plausible, the ICEMAN assessment suggested that the credibility of the observed effect modification was limited by reliance on aggregate data and inconsistency between observational and randomized evidence. Incorporation of insulin use data showed that baseline insulin therapy was more prevalent in participants with higher HbA1c levels across RCTs. In observational studies, insulin use was generally low, except for one study with a notably high proportion. Subgroup analysis of RCTs stratified by the proportion of insulin‐treated patients showed no meaningful evidence of effect modification by insulin use. Finally, a pooled analysis of three observational studies that directly examined the association between baseline HbA1c and DKA risk in SGLT‐2i users confirmed that individuals with higher HbA1c levels faced a greater risk of DKA than those with lower HbA1c. The GRADE certainty of the evidence ranged from high to low. Together, these findings suggest that poor baseline glycemic control may amplify the risk of DKA in patients with T2D treated with SGLT‐2is (graphical abstract), particularly in real‐world clinical practice.

To our knowledge, this is the first systematic review and meta‐analysis specifically designed to examine the interrelationship between baseline HbA1c levels, SGLT‐2i use and the risk of DKA in adults with T2D. Unlike prior studies, we employed two complementary approaches: (i) assessing whether baseline HbA1c modifies the strength or direction of the association between SGLT‐2i use and DKA, and (ii) determining whether higher baseline HbA1c is independently associated with an increased risk of DKA among SGLT‐2i users. While the cardio‐kidney benefits of SGLT‐2is across HbA1c subgroups have been explored extensively in CVOTs [14, 36], similar analyses focused on safety outcomes such as DKA have been lacking. A few related studies warrant comparison. Sridharan and colleagues [37] conducted a network meta‐analysis and meta‐regression assessing the risk of SGLT‐2i–induced DKA and potential modifiers. They found no significant effect modification by HbA1c levels. However, their findings may be limited by the inclusion of several studies with small sample sizes and a high proportion of zero DKA events in the treatment arm [37]—conditions that restrict the reliability of stratified analyses and meta‐regression. In a large real‐world comparative effectiveness study involving over 87 000 adults with T2D, D'Andrea and colleagues reported a significantly increased risk of DKA with SGLT‐2i versus DPP‐4i therapy, irrespective of HbA1c level [38]. While formal interaction testing did not identify statistically significant effect modification, subgroup analyses showed a two‐fold increase in DKA risk among individuals with HbA1c > 9% (HR = 2.06), compared with more modest increases in those with HbA1c < 7.5% (HR = 1.49) and 7.5%–9% (HR = 1.27) [38]. These findings suggest that the overall increased risk (HR = 1.73) may have been driven largely by individuals with poor glycemic control. Compared with these studies, our review benefits from stratified and meta‐regression analyses based on large studies and a dual focus on both observational and trial‐based evidence. Our findings add clarity to a previously underexplored question and offer novel insights into how baseline glycemic status may influence the safety profile of SGLT‐2is.

The increased risk of DKA observed among SGLT‐2i users with high baseline HbA1c is biologically plausible. SGLT‐2is promote glycosuria by inhibiting renal glucose reabsorption, which lowers plasma glucose levels independently of insulin [39]. While this improves glycemic control, it may also mask hyperglycemia and increase ketogenesis, particularly in individuals with underlying insulin deficiency.

The glycosuria induced by SGLT‐2is leads to osmotic diuresis and volume depletion—both of which are known DKA triggers [40]. This effect may be more pronounced in patients with poorly controlled diabetes, as urinary glucose excretion is greater in those with elevated HbA1c. Additionally, SGLT‐2is increase the risk of genitourinary infections [41], which are common DKA precipitants. In individuals with high HbA1c, insulin requirements are often higher and relative insulin deficiency, especially if insulin doses are reduced, can further increase the risk of ketosis [6]. Our analysis showed that baseline insulin use was more prevalent in trial participants with high HbA1c levels, supporting the hypothesis that these individuals may have greater insulin dependency. However, stratified analysis by insulin use did not reveal significant effect modification, suggesting that insulin therapy alone does not fully explain the observed HbA1c‐DKA relationship, though it may contribute to underlying susceptibility. Together, these mechanisms may explain the stronger association between SGLT‐2i use and DKA risk in those with poor glycemic control.

The findings of this review highlight important clinical considerations for the use of SGLT‐2is in the management of T2D. Specifically, the increased risk of DKA observed among individuals with higher baseline HbA1c suggests that poor glycemic control may amplify the adverse effects of SGLT‐2is (graphical abstract), particularly under certain stress conditions or physiological vulnerabilities. Clinicians should exercise caution when initiating SGLT‐2i therapy in patients with elevated HbA1c levels above 8.3%. Importantly, these findings should not be interpreted as a contraindication to SGLT‐2i use in individuals with poor glycaemic control, but rather as a signal to reinforce careful patient selection, education and monitoring to safely realize their substantial cardio‐kidney benefits. Individualized risk assessment is essential, taking into account not only glycemic status but also the presence of additional DKA risk factors such as recent infections, intercurrent illness, insulin deficiency or dose reductions, dehydration and psychological or physical stress. While SGLT‐2is offer clear cardiovascular and kidney benefits, these must be carefully weighed against the potential risks in patients with uncontrolled diabetes. Enhanced patient education, close clinical monitoring and adherence to sick‐day rules are crucial to safely maximizing the therapeutic benefits of SGLT‐2is in high‐risk populations.

## 5 Strengths and Limitations

This study has several notable strengths. It is the first systematic review and meta‐analysis to specifically examine the interrelationship between baseline HbA1c, SGLT‐2i use and the risk of DKA in adults with T2D, using two complementary analytical approaches. The inclusion of only studies with large sample sizes (*n* > 500) enhanced the statistical power and reliability of subgroup analyses. Additionally, the integration of both observational cohort studies and RCTs strengthens the generalizability and robustness of the findings across study designs and clinical settings. Importantly, we identified that several included studies were based on overlapping data sources. To mitigate the risk of double counting participants, we conducted a comprehensive evaluation to ensure that only non‐overlapping datasets were included in pooled analyses. This step minimized potential bias arising from duplication of patient populations and enhanced the accuracy of our effect estimates. We applied the ICEMAN framework to assess the credibility of the observed effect modification by baseline HbA1c. However, this study is not without limitations. The range of baseline HbA1c values across included studies, particularly in CVOTs, was relatively narrow. This constrained our ability to apply more clinically meaningful or granular HbA1c categories and may have reduced our sensitivity to detect variations in DKA risk across a broader spectrum of glycaemic control. Future analyses using pooled individual participant data could provide greater precision and allow for detailed exploration of this relationship. In observational settings, elevated baseline HbA1c may also reflect unmeasured insulin deficiency, treatment non‐adherence, or intercurrent illness, which could contribute to residual confounding and partially explain the stronger effect modification observed compared with randomized trials. In addition, due to limited reporting and aggregate‐level data, we were unable to formally evaluate whether the risk of DKA was disproportionately higher among insulin‐treated patients with elevated baseline HbA1c. This restricted our ability to assess potential synergistic effects between poor glycaemic control and insulin dependence on DKA risk following SGLT‐2i initiation. While a number of other studies were potentially eligible for inclusion, they did not report baseline HbA1c levels. We reached out to corresponding authors of these studies to request the missing data, but none responded, thereby limiting the comprehensiveness of our analysis. Given the limitations and the findings of the ICEMAN assessment, the overall results should be interpreted as hypothesis‐generating and warrant confirmation in individual participant data analyses.

## 6 Conclusions

Aggregate findings from both observational studies and RCTs suggest that individuals with higher baseline HbA1c levels may be at increased risk of DKA when treated with SGLT‐2is, compared to those with better glycemic control. These findings underscore the importance of individualized patient assessment prior to initiating SGLT‐2i therapy. Clinicians should remain vigilant, particularly in patients with poorly controlled diabetes, and ensure that DKA risk factors are addressed. While SGLT‐2is offer substantial cardio‐kidney benefits, careful patient selection and monitoring are essential to minimize rare but serious adverse events such as DKA. Future research should aim to refine our understanding of glycemia‐related risk thresholds and to establish evidence‐based strategies for mitigating DKA risk in high‐risk subgroups.

## Author Contributions

**Samuel Seidu:** study design, data interpretation, writing. **Setor K. Kunutsor:** study design, literature search, data collection, analysis, data interpretation, writing. **Erwin Taguiam:** study design, literature search, data interpretation and writing. **Kamlesh Khunti:** study design, data interpretation and writing.

## Funding

This work was supported by the National Institute for Health Research (NIHR) Applied Research Collaboration East Midlands (ARC EM).

## Ethics Statement

The authors have nothing to report.

## Conflicts of Interest

Samuel Seidu is in receipt of speaker honoraria from AstraZeneca, Boehringer Ingelheim, Janssen, Lilly, MSD, Abbott, Novo Nordisk, SB Communications, OmniaMed Communications, Roche, Napp Pharmaceuticals, NB Medical and Amgen; advisory board honoraria from AstraZeneca, Lilly, Boehringer Ingelheim, Janssen, Abbott, MSD, Novo Nordisk, Takeda and Sanofi; educational grants from Boehringer Ingelheim, Lilly, Novo Nordisk and Takeda; and conference registration and subsistence from Boehringer Ingelheim, Janssen, Lilly, Novo Nordisk, Abbott and Takeda. K.K. has acted as a consultant, speaker, or received grants for investigator‐initiated studies for Abbott, Astra Zeneca, Bayer, Hikma, Novo Nordisk, Sanofi‐Aventis, Servier, Lilly and Merck Sharp & Dohme; Boehringer Ingelheim, Oramed Pharmaceuticals, Pfizer, Roche, Daiichi‐Sankyo, Applied Therapeutics, Embecta, Nestle Health Science and Adelphi. S.K.K. and E.T. have no conflicts. Professor Samuel Seidu is the guarantor of this work and, as such, had full access to all the data in the study and takes responsibility for the integrity of the data and the accuracy of the data analysis.

## Supporting information


**Supporting Information: S1** PRISMA checklist.
**Supporting Information: S2** MOOSE checklist.
**Supporting Information: S3** Literature search strategy.
**Supporting Information: S4** PRISMA‐S checklist.
**Supporting Information: S5** Risk of bias assessment for observational cohort studies (ROBINS‐I v2).
**Supporting Information: S6** Risk of bias assessment for RCTs (ROB2).
**Supporting Information: S7** SGLT‐2is and DKA risk stratified by the proportion of insulin‐treated patients in the SGLT‐2i group in randomized controlled trials.
**Supporting Information: S8** Association between HbA1c and DKA risk among SGLT‐2i users with T2D.
**Supporting Information: S9** ICEMAN assessment of the credibility of effect modification by baseline HbA1c (high vs. low) on the association between SGLT‐2i use and DKA risk.
**Supporting Information: S10** GRADE summary of findings (observational cohort studies).
**Supporting Information: S11** GRADE summary of findings (randomized controlled trials).

## Data Availability

The corresponding author had full access to all the data in the study and takes responsibility for the integrity of the data and the accuracy of the data analysis. This study is based on data from published articles.
