# Supplementary material for: Interrelationship Between Baseline HbA1c, SGLT‐2 Inhibitor Use and Risk of Diabetic Ketoacidosis in Adults With Type 2 Diabetes: A Systematic Review and Meta‐Analysis
Source: Diabetes Obes Metab. 2026 Apr 7;28(6):5217–28. doi: 10.1111/dom.70728 (PMC13146127; doi:10.1111/dom.70728)
Supplement: Supplementary file 1 — Supporting Information: S1 PRISMA checklist. Supporting Information: S2 MOOSE checklist. Supporting Information: S3 Literature search strategy. Supporting Information: S4 PRISMA‐S checklist. Supporting Information: S5 Risk of bias assessment for observational cohort studies (ROBINS‐I v2). Supporting Information: S6 Risk of bias assessment for RCTs (ROB2). Supporting Information: S7 SGLT‐2is and DKA risk stratified by the proportion of insulin‐treated patients in the SGLT‐2i group in randomized controlled trials. Supporting Information: S8 Association between HbA1c and DKA risk among SGLT‐2i users with T2D. Supporting Information: S9 ICEMAN assessment of the credibility of effect modification by baseline HbA1c (high vs. low) on the association between SGLT‐2i use and DKA risk. Supporting Information: S10 GRADE summary of findings (observational cohort studies). Supporting Information: S11 GRADE summary of findings (randomized controlled trials). [file DOM-28-5217-s001.docx]

**SUPPLEMENTARY MATERIAL**

| **Supplementary Material 1** | PRISMA checklist |
| --- | --- |
| **Supplementary Material 2** | MOOSE Checklist |
| **Supplementary Material 3** | Literature search strategy |
| **Supplementary Material 4** | PRISMA-S Checklist |
| **Supplementary Material 5** | Risk of bias assessment for observational cohort studies (ROBINS-I v2) |
| **Supplementary Material 6** | Risk of bias assessment for RCTs (ROB2) |
| **Supplementary Material 7** | SGLT-2is and DKA risk stratified by the proportion of insulin-treated patients in the SGLT-2i group in randomized controlled trials |
| **Supplementary Material 8** | Association between HbA1c and DKA risk among SGLT-2i users with T2D |
| **Supplementary Material 9** | ICEMAN assessment of the credibility of effect modification by baseline HbA1c (high vs low) on the association between SGLT-2i use and DKA risk |
| **Supplementary Material 10** | GRADE summary of findings (Observational cohort studies) |
| **Supplementary Material 11** | GRADE summary of findings (Randomized controlled trials) |

**Supplementary Material 1:** PRISMA checklist

| **Section/topic** | **Item No** | **Checklist item** | **Reported on page No** |
| --- | --- | --- | --- |
| **Title** | | | |
| Title | 1 | Identify the report as a systematic review, meta-analysis, or both | 1 |
| **Abstract** | | | |
| Structured summary | 2 | Provide a structured summary including, as applicable, background, objectives, data sources, study eligibility criteria, participants, interventions, study appraisal and synthesis methods, results, limitations, conclusions and implications of key findings, systematic review registration number | 2 |
| **Introduction** | | | |
| Rationale | 3 | Describe the rationale for the review in the context of what is already known | Introduction |
| Objectives | 4 | Provide an explicit statement of questions being addressed with reference to participants, interventions, comparisons, outcomes, and study design (PICOS) | Introduction |
| **Methods** | | | |
| Protocol and registration | 5 | Indicate if a review protocol exists, if and where it can be accessed (such as web address), and, if available, provide registration information including registration number | Methods |
| Eligibility criteria | 6 | Specify study characteristics (such as PICOS, length of follow-up) and report characteristics (such as years considered, language, publication status) used as criteria for eligibility, giving rationale | Methods |
| Information sources | 7 | Describe all information sources (such as databases with dates of coverage, contact with study authors to identify additional studies) in the search and date last searched | Methods |
| Search | 8 | Present full electronic search strategy for at least one database, including any limits used, such that it could be repeated | Supplementary Material 3 |
| Study selection | 9 | State the process for selecting studies (that is, screening, eligibility, included in systematic review, and, if applicable, included in the meta-analysis) | Methods |
| Data collection process | 10 | Describe method of data extraction from reports (such as piloted forms, independently, in duplicate) and any processes for obtaining and confirming data from investigators | Methods |
| Data items | 11 | List and define all variables for which data were sought (such as PICOS, funding sources) and any assumptions and simplifications made | Methods |
| Risk of bias in individual studies | 12 | Describe methods used for assessing risk of bias of individual studies (including specification of whether this was done at the study or outcome level), and how this information is to be used in any data synthesis | Methods |
| Summary measures | 13 | State the principal summary measures (such as risk ratio, difference in means). | Methods |
| Synthesis of results | 14 | Describe the methods of handling data and combining results of studies, if done, including measures of consistency (such as I^2^ statistic) for each meta-analysis | Methods |
| Risk of bias across studies | 15 | Specify any assessment of risk of bias that may affect the cumulative evidence (such as publication bias, selective reporting within studies) | Methods |
| Additional analyses | 16 | Describe methods of additional analyses (such as sensitivity or subgroup analyses, meta-regression), if done, indicating which were pre-specified | Methods |
| **Results** | | | |
| Study selection | 17 | Give numbers of studies screened, assessed for eligibility, and included in the review, with reasons for exclusions at each stage, ideally with a flow diagram | Results, Figure 1 |
| Study characteristics | 18 | For each study, present characteristics for which data were extracted (such as study size, PICOS, follow-up period) and provide the citations | Results, Table 1 |
| Risk of bias within studies | 19 | Present data on risk of bias of each study and, if available, any outcome-level assessment (see item 12). | Results, Supplementary Materials 5-6 |
| Results of individual studies | 20 | For all outcomes considered (benefits or harms), present for each study (a) simple summary data for each intervention group and (b) effect estimates and confidence intervals, ideally with a forest plot |  |
| Synthesis of results | 21 | Present results of each meta-analysis done, including confidence intervals and measures of consistency | Results, Figures 1-4 |
| Risk of bias across studies | 22 | Present results of any assessment of risk of bias across studies (see item 15) | Results |
| Additional analysis | 23 | Give results of additional analyses, if done (such as sensitivity or subgroup analyses, meta-regression) (see item 16) | Results |
| **Discussion** | | | |
| Summary of evidence | 24 | Summarise the main findings including the strength of evidence for each main outcome; consider their relevance to key groups (such as health care providers, users, and policy makers) | Discussion |
| Limitations | 25 | Discuss limitations at study and outcome level (such as risk of bias), and at review level (such as incomplete retrieval of identified research, reporting bias) | Discussion |
| Conclusions | 26 | Provide a general interpretation of the results in the context of other evidence, and implications for future research | Discussion |
| **Funding** | | | |
| Funding | 27 | Describe sources of funding for the systematic review and other support (such as supply of data) and role of funders for the systematic review | After Discussion |

**Supplementary Material 2:** MOOSE checklist

| **Criteria** | | **Brief description of how the criteria were handled in the review** |
| --- | --- | --- |
| **Reporting of background** | |  |
| √ | Problem definition | There is ongoing uncertainty about whether initiating sodium–glucose co-transporter 2 inhibitor (SGLT-2i) therapy in individuals with type 2 diabetes (T2D) who have elevated baseline HbA1c levels may lead to an additive—or potentially synergistic—increase in the risk of diabetic ketoacidosis (DKA). |
| √ | Hypothesis statement | 1. Higher baseline HbA1c is associated with an increased risk of DKA in individuals with T2D who are treated with SGLT-2is.  2. Baseline HbA1c modifies the association between SGLT-2i use and DKA risk in T2D patients. |
| √ | Description of study outcomes | DKA |
| √ | Type of exposure | HbA1c and SGLT-2i |
| √ | Type of study designs used | Observational cohort studies and randomized controlled trials |
| √ | Study population | Adults (≥18 years) with type 2 diabetes or adults with type 2 diabetes using SGLT-2is |
| **Reporting of search strategy should include** | |  |
| √ | Qualifications of searchers | Setor K. Kunutsor, PhD; Erwin Taguiam, MSc; Samuel Seidu, MD |
| √ | Search strategy, including time period included in the synthesis and keywords | Time period: from inception to January 2026  The detailed search strategy can be found in Supplementary Material 3 |
| √ | Databases and registries searched | MEDLINE, Embase, CENTRAL |
| √ | Search software used, name and version, including special features | OvidSP was used to search Embase and MEDLINE  EndNote used to manage references; Covidence used for screening |
| √ | Use of hand searching | We searched bibliographies of retrieved papers |
| √ | List of citations located and those excluded, including justifications | Details of the literature search process are outlined in the flow chart. The citation list for excluded studies is available on request. |
| √ | Method of addressing articles published in languages other than English | Not applicable |
| √ | Method of handling abstracts and unpublished studies | Excluded |
| √ | Description of any contact with authors | Contact with investigators but no response |
| **Reporting of methods should include** | |  |
| √ | Description of relevance or appropriateness of studies assembled for assessing the hypothesis to be tested | Detailed inclusion and exclusion criteria are described in the Methods section. |
| √ | Rationale for the selection and coding of data | Data extracted from each of the studies were relevant to the population characteristics, study design, intervention/exposure, and outcome. |
| √ | Assessment of confounding | We required observational studies implement 1:1 propensity score matching or equivalent adjustment strategies. |
| √ | Assessment of study quality, including blinding of quality assessors; stratification or regression on possible predictors of study results | The Cochrane Risk of Bias in Non-randomised Studies – of Interventions (ROBINS-I) tool was used to assess the risk of bias within individual observational studies; for randomized controlled trials, we used the Cochrane Collaboration’s Risk of Bias tool |
| √ | Assessment of heterogeneity | Heterogeneity of the studies was quantified with I^2^ statistic that provides the relative amount of variance of the summary effect due to the between-study heterogeneity |
| √ | Description of statistical methods in sufficient detail to be replicated | Description of methods of meta-analyses, sensitivity analyses, meta-regression and assessment of publication bias are detailed in the methods. We performed random effects meta-analysis with Stata 18. |
| √ | Provision of appropriate tables and graphics | Table 1; Figures 1-4 |
| **Reporting of results should include** | |  |
| √ | Graph summarizing individual study estimates and overall estimate | Figures 1-4 |
| √ | Table giving descriptive information for each study included | Table 1 |
| √ | Results of sensitivity testing | Not applicable |
| √ | Indication of statistical uncertainty of findings | 95% confidence intervals were presented with all summary estimates, I^2^ values and results of sensitivity analyses |
| **Reporting of discussion should include** | |  |
| √ | Quantitative assessment of bias | The systematic review is limited in scope, as it involves published data. Individual participant data is needed. Limitations have been discussed. |
| √ | Justification for exclusion | All studies were excluded based on the pre-defined inclusion criteria in methods section. |
| √ | Assessment of quality of included studies | Brief discussion included in ‘Methods’ section |
| **Reporting of conclusions should include** | |  |
| √ | Consideration of alternative explanations for observed results | Discussion |
| √ | Generalization of the conclusions | Discussed in the context of the results. |
| √ | Guidelines for future research | We recommend individual participant data meta-analysis |
| √ | Disclosure of funding source | In “Acknowledgement” section |

**Supplementary Material 3:** Literature search strategy

| **Ovid MEDLINE** |
| --- |
| 1  exp Diabetes Mellitus, Type 2/ or Diabetes Mellitus, Type 2.mp. (195387)  2  type 2 diabetes.mp. (199738)  3  T2DM.mp. (43051)  4  exp Sodium-Glucose Transporter 2 Inhibitors/ (8949)  5  SGLT-2.mp. (2310)  6  SGLT2.mp. (8766)  7  exp Canagliflozin/ (1212)  8  Dapagliflozin.mp. (3944)  9  Empagliflozin.mp. (3969)  10  Ertugliflozin.mp. (347)  11  Sotagliflozin.mp. (275)  12  exp Diabetic Ketoacidosis/ (8098)  13  ketoacidosis.mp. (14515)  14  DKA.mp. (3969)  15  Glycated Hemoglobin A.mp. or exp Glycated Hemoglobin/ (48107)  16  HbA1c.mp. (56139)  17  glycosylated hemoglobin.mp. (10063)  18  1 or 2 or 3 (267155)  19  4 or 5 or 6 or 7 or 8 or 9 or 10 or 11 (16429)  20  12 or 13 or 14 (14773)  21  15 or 16 or 17 (83184)  22  18 and 19 and 20 and 21 (109)  23  limit 22 to (humans and "remove preprint records") (90) |
|  |
| **Ovid Embase** |
| 1  exp non insulin dependent diabetes mellitus/ or Diabetes Mellitus, Type 2.mp. (432584)  2  type 2 diabetes.mp. (316836)  3  T2DM.mp. (71740)  4  Sodium-Glucose Transporter 2 Inhibitors.mp. or exp sodium glucose cotransporter 2 inhibitor/ (41544)  5  exp dapagliflozin/ or exp empagliflozin/ or SGLT2.mp. (28070)  6  SGLT-2.mp. (4195)  7  exp canagliflozin/ (7206)  8  exp ertugliflozin/ (1643)  9  exp sotagliflozin/ (1218)  10  exp diabetic ketoacidosis/ (21601)  11  DKA.mp. (9088)  12  exp ketoacidosis/ (32967)  13  exp hemoglobin A1c/ or Glycated Hemoglobin A.mp. or exp glycated hemoglobin/ (219068)  14  HbA1c.mp. (121141)  15  glycosylated hemoglobin.mp. (14313)  16  1 or 2 or 3 (485984)  17  4 or 5 or 6 or 7 or 8 or 9 (43720)  18  10 or 11 or 12 (34878)  19  13 or 14 or 15 (228472)  20  16 and 17 and 18 and 19 (1106)  21  limit 20 to (human and "remove clinical trial (clinicaltrials.gov) records" and "remove medline records" and "remove preprint records") (478) |

**Supplementary Material 4:** PRISMA-S Checklist

| **Section/topic** | **#** | **Checklist item** | **Location(s) Reported** |
| --- | --- | --- | --- |
| **INFORMATION SOURCES AND METHODS** | | | |
| Database name | 1 | Name each individual database searched, stating the platform for each. | Methods |
| Multi-database searching | 2 | If databases were searched simultaneously on a single platform, state the name of the platform, listing all of the databases searched. | N/A |
| Study registries | 3 | List any study registries searched. | N/A |
| Online resources and browsing | 4 | Describe any online or print source purposefully searched or browsed (e.g., tables of contents, print conference proceedings, web sites), and how this was done. | N/A |
| Citation searching | 5 | Indicate whether cited references or citing references were examined, and describe any methods used for locating cited/citing references (e.g., browsing reference lists, using a citation index, setting up email alerts for references citing included studies). | Methods |
| Contacts | 6 | Indicate whether additional studies or data were sought by contacting authors, experts, manufacturers, or others. | N/A |
| Other methods | 7 | Describe any additional information sources or search methods used. | N/A |
| **SEARCH STRATEGIES** | | | |
| Full search strategies | 8 | Include the search strategies for each database and information source, copied and pasted exactly as run. | Supplementary Material 3 |
| Limits and restrictions | 9 | Specify that no limits were used, or describe any limits or restrictions applied to a search (e.g., date or time period, language, study design) and provide justification for their use. | Methods |
| Search filters | 10 | Indicate whether published search filters were used (as originally designed or modified), and if so, cite the filter(s) used. | Methods |
| Prior work | 11 | Indicate when search strategies from other literature reviews were adapted or reused for a substantive part or all of the search, citing the previous review(s). | Methods |
| Updates | 12 | Report the methods used to update the search(es) (e.g., rerunning searches, email alerts). | N/A |
| Dates of searches | 13 | For each search strategy, provide the date when the last search occurred. | Methods |
| **PEER REVIEW** | | | |
| Peer review | 14 | Describe any search peer review process. | N/A |
| **MANAGING RECORDS** | | | |
| Total Records | 15 | Document the total number of records identified from each database and other information sources. | Supplementary Material 3 |
| Deduplication | 16 | Describe the processes and any software used to deduplicate records from multiple database searches and other information sources. | Methods |
|  |  |  |  |

**Supplementary Material 5:** Risk of bias assessment for observational cohort studies (ROBINS-I v2)

|  |  | **Risk of bias domains** | | | | | | |  |
| --- | --- | --- | --- | --- | --- | --- | --- | --- | --- |
| **Study** |  | D1 | D2 | D3 | D4 | D5 | D6 | Overall | |
|  | Fralick, 2021a | Moderate | Low | Low | Serious | Low | Low | Serious | |
|  | Patorno, 2021 | Moderate | Low | Low | Serious | Low | Low | Serious | |
|  | Gudemann, 2024 | Moderate | Low | Low | Moderate | Low | Low | Moderate | |
|  | McGurnaghan, 2019 | Serious | Low | Low | Moderate | Low | Low | Serious | |
|  | Htoo, 2024 | Moderate | Low | Low | Moderate | Low | Low | Moderate | |
|  | Lugner, 2021 | Moderate | Low | Low | Low | Low | Low | Moderate | |
|  | Chen, 2025 | Moderate | Low | Low | Moderate | Low | Low | Moderate | |
|  | Goh, 2022 | Moderate | Low | Moderate | Moderate | Low | Low | Moderate | |
|  | Edmonston, 2024 | Moderate | Low | Low | Low | Low | Low | Moderate | |
|  | Suzuki, 2025 | Moderate | Low | Low | Low | Low | Low | Moderate | |
|  | Pan, 2024 | Moderate | Low | Moderate | Moderate | Low | Low | Moderate | |
|  | Yen, 2024 | Moderate | Low | Low | Moderate | Low | Low | Moderate | |
|  | Fralick, 2021b | Moderate | Low | Moderate | Serious | Low | Low | Serious | |
|  | Tsur, 2025 | Moderate | Low | Low | Moderate | Low | Low | Moderate | |

| **Legend:** |  |  | **Judgment:** |
| --- | --- | --- | --- |
| D1 | Risk of bias due to confounding |  | Low |
| D2 | Risk of bias in classification of interventions |  | Moderate |
| D3 | Risk of bias in selection of participants into the study (or into the analysis) |  | Serious |
| D4 | Risk of bias due to missing data |  |  |
| D5 | Risk of bias arising from measurement of the outcome |  |  |
| D6 | Risk of bias in selection of the reported result |  |  |

*, risk of bias could not be assessed for Jing, 2024 because the full paper was not available.

**Supplementary Material 6:** Risk of bias assessment for RCTs (ROB2)

**
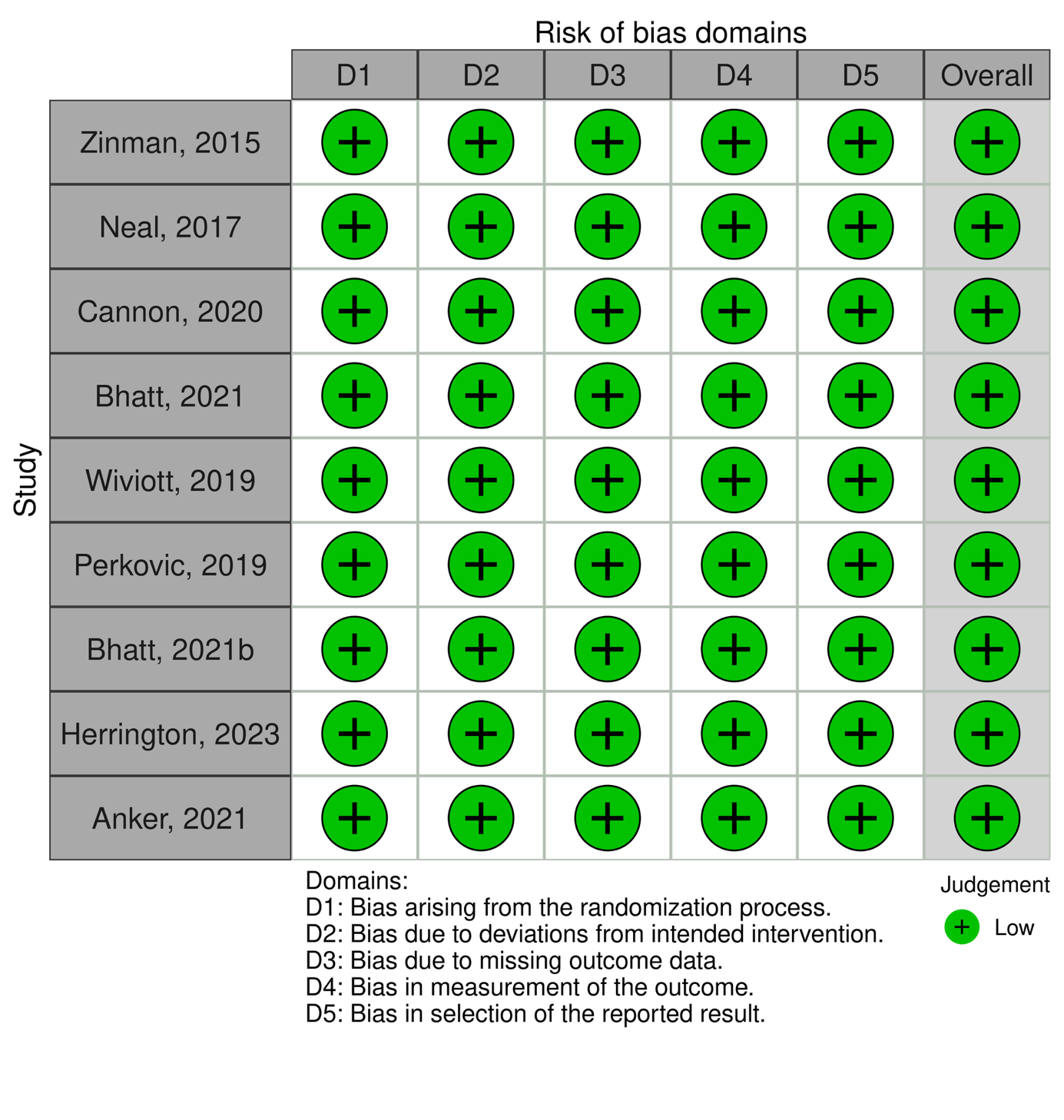
**

**Supplementary Material 7:** SGLT-2is and DKA risk stratified by the proportion of insulin-treated patients in the SGLT-2i group in randomized controlled trials

CI, confidence interval (bars); DKA, diabetic ketoacidosis; RR, risk ratio; SGLT-2i, sodium–glucose co-transporter 2 inhibitor

**Supplementary Material 8:** Association between HbA1c and DKA risk among SGLT-2i users with T2D

CI, confidence interval (bars); DKA, diabetic ketoacidosis; HbA1c, glycated haemoglobin; RR, risk ratio; SGLT-2i, sodium–glucose co-transporter 2 inhibitor; T2D, type 2 diabetes

**Supplementary Material 9.** ICEMAN assessment of the credibility of effect modification by baseline HbA1c (high vs low) on the association between SGLT-2i use and DKA risk

Effect modifier: Baseline HbA1c category (high vs low; study-level mean/median split)
Outcome: Diabetic ketoacidosis (DKA)
Effect estimate: Risk ratio (random-effects model)
Interaction test: Random-effects meta-regression (study-level)

| **ICEMAN Domain / Question** | **Assessment** | **Rationale** |
| --- | --- | --- |
| **Observational Studies** |  |  |
| 1. Is effect modification based on between-study comparisons? | Completely between | Subgroups were defined using study-level HbA1c averages, not individual-level interaction estimates. |
| 2. For within-study comparisons, is the effect modification similar from study to study? | Not applicable | No within-study comparison |
| 3. For between-study comparisons, is the number of studies large? | Rather large | 5-9 in smallest subgroup |
| 4. Was the direction of effect modification specified a priori? | Definitely yes | Greater DKA risk with higher HbA1c is biologically plausible. |
| 5. Does a test for interaction suggest that chance is an unlikely explanation of the apparent effect modification? | Chance may not explain | Stratified pooled effects and meta-regression supported interaction in observational data. |
| 6. Did the authors test only a small number of effect modifiers or consider the number in their statistical analysis? | Unclear | 4-10 effect modifiers tested and number not considered in analysis |
| 7. Did the authors use a random effects model? | Definitely yes | Random effects model used |
| 8. If the effect modifier is a continuous variable, were arbitrary cut points avoided? | Unclear | Analysis based on post-hoc cut points using the distribution of the data. |
| 9. Could bias or confounding explain findings? | Yes | Residual confounding and ecological bias are possible in observational data. |
| **Overall ICEMAN credibility rating** | **Moderate** | **Biologically plausible and statistically supported, but limited by aggregate-level analysis and potential confounding.** |
|  |  |  |
| **RCTs** |  |  |
| 1. Is effect modification based on between-trial comparisons? | Completely between | Categorization based on trial-level HbA1c means, not individual-level interaction testing. |
| 2. For within-trial comparisons, is the effect modification similar from trial to trial? | Not applicable | No within-RCT comparison |
| 3. For between-trial comparisons, is the number of trials large? | Rather small or unclear | 3-4 in smallest subgroup |
| 4. Was the direction of effect modification specified a priori? | Definitely yes | Greater DKA risk with higher HbA1c is biologically plausible. |
| 5. Does a test for interaction suggest that chance is an unlikely explanation of the apparent effect modification? | Chance a likely explanation | Low event counts increase the likelihood of chance findings. Meta-regression did not demonstrate statistically significant interaction in RCTs. |
| 6. Did the authors test only a small number of effect modifiers or consider the number in their statistical analysis? | Unclear | 4-10 effect modifiers tested and number not considered in analysis |
| 7. Did the authors use a random effects model? | Definitely yes | Random effects model used |
| 8. If the effect modifier is a continuous variable, were arbitrary cut points avoided? | Unclear | Analysis based on post-hoc cut points using the distribution of the data. |
| **Overall ICEMAN credibility rating** | **Low** | **Biological plausibility exists but limited statistical support and aggregate-level subgrouping reduce credibility.** |

ICEMAN = Instrument to assess the Credibility of Effect Modification Analyses. Assessments reflect subgroup analyses based primarily on aggregate (study-level) baseline HbA1c values rather than within-study individual-level interaction effects.

DKA, diabetic ketoacidosis; SGLT-2i, sodium–glucose co-transporter 2 inhibitor

**Supplementary Material 10:** GRADE summary of findings (Observational cohort studies)

| **SGLT-2i compared to Non-SGLT-2i in DKA (Observational Studies)** | | | | | |
| --- | --- | --- | --- | --- | --- |
| **Outcomes** | **№ of participants (studies) Follow-up** | **Certainty of the evidence (GRADE)** | **Relative effect (95% CI)** | **Anticipated absolute effects** | |
|  |  |  |  | **Risk with Non-SGLT-2i** | **Risk difference with SGLT-2i** |
| SGLT-2is and DKA risk in higher HbA1c group | 821562 (5 non-randomised studies) | ⨁⨁⨁◯ Moderate^a^ | **RR 1.63** (1.46 to 1.81) | 2 per 1,000 | **1 more per 1,000** (1 more to 1 more) |
| SGLT-2is and DKA risk in lower HbA1c group | 447443 (7 non-randomised studies) | ⨁⨁◯◯ Low^a,b^ | **RR 1.10** (0.80 to 1.51) | 7 per 1,000 | **1 more per 1,000** (1 fewer to 4 more) |
| ***The risk in the intervention group** (and its 95% confidence interval) is based on the assumed risk in the comparison group and the **relative effect** of the intervention (and its 95% CI).  **CI:** confidence interval; **RR:** risk ratio | | | | | |
| **GRADE Working Group grades of evidence** **High certainty:** we are very confident that the true effect lies close to that of the estimate of the effect. **Moderate certainty:** we are moderately confident in the effect estimate: the true effect is likely to be close to the estimate of the effect, but there is a possibility that it is substantially different. **Low certainty:** our confidence in the effect estimate is limited: the true effect may be substantially different from the estimate of the effect. **Very low certainty:** we have very little confidence in the effect estimate: the true effect is likely to be substantially different from the estimate of effect. | | | | | |

#### Explanations

a. At serious risk of bias in at least one study.

b. I-squared of 80%.

**Supplementary Material 11:** GRADE summary of findings (Randomized controlled trials)

| **SGLT-2i compared to Placebo in DKA (Randomized controlled trials)** | | | | | |
| --- | --- | --- | --- | --- | --- |
| **Outcomes** | **№ of participants (studies) Follow-up** | **Certainty of the evidence (GRADE)** | **Relative effect (95% CI)** | **Anticipated absolute effects** | |
|  |  |  |  | **Risk with Placebo** | **Risk difference with SGLT-2i** |
| SGLT-2is and DKA risk in higher HbA1c group | 32145 (6 RCTs) | ⨁⨁⨁⨁ High^a^ | **RR 2.37** (1.44 to 3.90) | 2 per 1,000 | **2 more per 1,000** (1 more to 5 more) |
| SGLT-2is and DKA risk in lower HbA1c group | 26616 (4 RCTs) | ⨁⨁⨁◯ Moderate^a^ | **RR 2.01** (0.84 to 4.79) | 5 per 1,000 | **5 more per 1,000** (1 fewer to 18 more) |
| ***The risk in the intervention group** (and its 95% confidence interval) is based on the assumed risk in the comparison group and the **relative effect** of the intervention (and its 95% CI).  **CI:** confidence interval; **RR:** risk ratio | | | | | |
| **GRADE Working Group grades of evidence** **High certainty:** we are very confident that the true effect lies close to that of the estimate of the effect. **Moderate certainty:** we are moderately confident in the effect estimate: the true effect is likely to be close to the estimate of the effect, but there is a possibility that it is substantially different. **Low certainty:** our confidence in the effect estimate is limited: the true effect may be substantially different from the estimate of the effect. **Very low certainty:** we have very little confidence in the effect estimate: the true effect is likely to be substantially different from the estimate of effect. | | | | | |

#### Explanations

a. Wide 95% CI
